# Performance of the PEdiatric Logistic Organ Dysfunction-2 score in critically ill children requiring plasma transfusions

**DOI:** 10.1186/s13613-016-0197-6

**Published:** 2016-10-06

**Authors:** Oliver Karam, Pierre Demaret, Alain Duhamel, Alison Shefler, Philip C. Spinella, Simon J. Stanworth, Marisa Tucci, Stéphane Leteurtre, for the PlasmaTV investigators, Warwick Butt, Carmel Delzoppo, Kym Bain, Simon Erickson, Nathan Smalley, Tavey Dorofaeff, Debbie Long, Greg Wiseman, Stéphan Clénent de Cléty, Caroline Berghe, Annick de Jaeger, Pierre Demaret, Marc Trippaerts, Ariane Willems, Shancy Rooze, Jozef De Dooy, Elaine Gilfoyle, Lynette Wohlgemuth, Marisa Tucci, Mariana Dumitrascu, Davinia Withington, Julia Hickey, Karen Choong, Lois Sanders, Gavin Morrison, Janice Tijssen, David Wensley, Gordon Krahn, Marc-Andre Dugas, Louise Gosselin, Miriam Santschi, Bettina Von Dessauer, Nadia Ordenes, Arash Afshari, Lasse Hoegh Andersen, Jens Christian Nilsson, Mathias Johansen, Anne-Mette Baek Jensen, Santiago Campos Mino, Michelle Grunauer, Nicolas Joram, Nicolas Roullet-Renoleau, Etienne Javouhey, Fleur Cour-Andlauer, Aurélie Portefaix, Olivier Brissaud, Julie Guichoux, Valérie Payen, Pierre-Louis Léger, Mickael Afanetti, Guillaume Mortamet, Matthieu Maria, Audrey Breining, Pierre Tissieres, Aimée Dorkenoo, Anna Deho, Harry Steinherr, Filippia Nikolaou, Anna Camporesi, Federica Mario, Tatsuya Kawasaki, Shinya Miura, John Beca, Miriam Rea, Claire Sherring, Tracey Bushell, Gunnar Bentsen, Alexandra Dinis, Gabriela Pereira, Marisa Vieira, Marta Moniz, Saleh Alshehri, Manal Alasnag, Maria Pisarcikova, Iolanda Jordan, Joan Balcells, Antonio Perez-Ferrer, Jesús de Vicente Sánchez, Marta Vazquez Moyano, Antonio Morales Martinez, Jesus Lopez-Herce, Maria Jose Solana, Jose Carlos Flores González, Maria Teresa Alonso, Manuel Nieto Faza, Marie-Hélène Perez, Vivianne Amiet, Carsten Doell, Alice Bordessoule, Suzan Cochius-den Otter, Berber Kapitein, Martin Kneyber, Joe Brierley, Vanessa Rea, Stephen McKeever, Andrea Kelleher, Barney Scholefield, Anke Top, Nicola Kelly, Satnam Virdee, Peter Davis, Susan George, Kay C. Hawkins, Katie McCall, Victoria Brown, Kim Sykes, Richard Levin, Isobel MacLeod, Marie Horan, Petr Jirasek, David Inwald, Amina Abdulla, Sophie Raghunanan, Bob Taylor, Alison Shefler, Hannah Sparkes, Sheila Hanson, Katherine Woods, David Triscari, Kathy Murkowski, Caroline Ozment, Marie Steiner, Dan Nerheim, Amanda Galster, Renee Higgerson, LeeAnn Christie, Philip C. Spinella, Daniel Martin, Liz Rourke, Jennifer Muszynski, Lisa Steele, Samuel Ajizian, Michael C. McCrory, Kevin O’Brien, Christopher Babbitt, Erin Felkel, Glenn Levine, Edward J. Truemper, Machelle Zink, Marianne Nellis, Neal J. Thomas, Debbie Spear, Barry Markovitz, Jeff Terry, Rica Morzov, Vicki Montgomery, Andrew Michael, Melissa Thomas, Marcy Singleton, Dean Jarvis, Sholeen Nett, Douglas Willson, Michelle Hoot, Melania Bembea, Alvin Yiu, David McKinley, Elizabeth Scarlett, Jennifer Sankey, Minal Parikh, E. Vincent S. Faustino, Kelly Michelson, Jay Rilinger, Laura Campbell, Shira Gertz, Jill M. Cholette, Asumthia Jeyapalan, Margaret Parker, Scot Bateman, Amanda Johnson

**Affiliations:** 1 Pediatric Intensive Care Unit, Geneva University Hospital, Geneva, Switzerland; 2 Univ. Lille, EA 2694 - Santé Publique: épidémiologie et qualité des soins, 59000 Lille, France; 3 Pediatric Intensive Care Unit, CHC Liège, Liège, Belgium; 4 Department of Biostatistics, CHU Lille, 59000 Lille, France; 5 Pediatric Intensive Care Unit, Oxford University Hospitals, Oxford, UK; 6 Division of Critical Care, Department of Pediatrics, Washington University in St. Louis, St. Louis, MO USA; 7 NHS Blood and Transplant, John Radcliffe Hospital, Oxford, UK; 8 Pediatric Intensive Care Unit, CHU Sainte-Justine, Montreal, Canada; 9 Pediatric Intensive Care Unit, CHU Lille, 59000 Lille, France

**Keywords:** Plasma transfusion, Critical care, Children, Outcome, Multiple organ failure, Score

## Abstract

**Background:**

Organ dysfunction scores, based on physiological parameters, have been created to describe organ failure. In a general pediatric intensive care unit (PICU) population, the PEdiatric Logistic Organ Dysfunction-2 score (PELOD-2) score had both a good discrimination and calibration, allowing to describe the clinical outcome of critically ill children throughout their stay. This score is increasingly used in clinical trials in specific subpopulation. Our objective was to assess the performance of the PELOD-2 score in a subpopulation of critically ill children requiring plasma transfusions.

**Methods:**

This was an ancillary study of a prospective observational study on plasma transfusions over a 6-week period, in 101 PICUs in 21 countries. All critically ill children who received at least one plasma transfusion during the observation period were included. PELOD-2 scores were measured on days 1, 2, 5, 8, and 12 after plasma transfusion. Performance of the score was assessed by the determination of the discrimination (area under the ROC curve: AUC) and the calibration (Hosmer–Lemeshow test).

**Results:**

Four hundred and forty-three patients were enrolled in the study (median age and weight: 1 year and 9.1 kg, respectively). Observed mortality rate was 26.9 % (119/443). For PELOD-2 on day 1, the AUC was 0.76 (95 % CI 0.71–0.81) and the Hosmer–Lemeshow test was *p* = 0.76. The serial evaluation of the changes in the daily PELOD-2 scores from day 1 demonstrated a significant association with death, adjusted for the PELOD-2 score on day 1.

**Conclusions:**

In a subpopulation of critically ill children requiring plasma transfusion, the PELOD-2 score has a lower but acceptable discrimination than in an entire population. This score should therefore be used cautiously in this specific subpopulation.

## Background

Mortality is a frequent outcome in clinical trials in critically ill adults [1–5]. However, as mortality is lower in pediatric intensive care unit (PICU) patients [6–8], other outcome measures have been developed. Multiple organ dysfunction syndrome (MODS), frequently observed in PICU, is a good candidate marker of severity of illness because MODS is the main cause of death in adult ICU [9] and in PICU patients [7]. MODS scores can be used to assess the presence and severity of organ dysfunction on admission and throughout the stay [9].

In a general PICU population, the PEdiatric Logistic Organ Dysfunction-2 score (PELOD-2) and the daily PELOD-2 scores had both a good discrimination and calibration, allowing to describe the clinical outcome of critically ill children throughout their stay [8, 10].

Little is known regarding plasma use in children. Our recent international observational study shows that non-bleeding patients represent more than half of the critically ill children receiving plasma transfusions [11]. This marked heterogeneity in plasma transfusion patterns might be due to the absence of randomized controlled trials (RCTs) that could guide plasma transfusion strategies [12].

The first version of the PELOD score [7] has been used over the last few years as an outcome measure in studies in specific subpopulations, such as sepsis [13], hematopoietic stem cell transplant [14], acute respiratory dysfunction syndrome [15], extracorporeal life support [16], drowning [17], or seizure [18]. However, some authors have voiced their concern regarding using organ dysfunction scores in specific subpopulations [9].

Our hypothesis was that the PELOD-2 score would have the same performance in the subpopulation of patients receiving at least one plasma transfusion, validating its use as a surrogate outcome in a future RCT.

Our objective was to assess the performance of the PELOD-2 score in a subset of critically ill children requiring plasma transfusions [11] during their PICU stay.

## Methods

### Study sites and population

This is an ancillary study of a large point-prevalence study conducted in 101 PICUs in 21 countries. The complete methods have already been published elsewhere [11].

In brief, six 1-week periods were randomly predefined over six consecutive months (April to September 2014) for each study site. All critically ill children aged 3 days to 16 years old admitted to a participating PICU on one of the study days were considered eligible. Any eligible patient for whom at least one plasma transfusion was administered on any study day was included unless one of the exclusion criteria (i.e., plasmapheresis and gestational age less than 37 weeks at the time of PICU admission) was present. If a patient was readmitted within 24 h of PICU discharge, this was considered part of the same admission.

### Variable of interest

The primary variable of interest of this analysis is the daily PELOD-2 scores [8, 10]. This score evaluates five organ functions using ten items: neurologic (Glasgow coma score and pupillary reaction), cardiovascular (lactatemia, mean arterial pressure), renal (creatinine), respiratory (PaO_2_/FiO_2_ ratio, PaCO_2_, invasive ventilation), and hematologic (white blood cell count and platelets). Data were collected on days 1 (i.e., the day of first transfusion), 2, 5, 8, and 12. These time points were previously identified as the first optimal time points to estimate the daily PELOD-2 scores [8, 10].

As for previously published severity and MODS scores, the most abnormal value of each variable observed during each of these time points was considered to calculate the PELOD-2 score. No laboratory tests were performed solely to meet the needs of this research; as recommended, a non-collected value was considered normal [8].

We also collected demographic data and PICU mortality, our primary outcome, which was censored 28 days after the end of the enrollment period.

### Ethics approval

Ethics committees or boards at all 101 sites approved this study.

### Statistical analysis

Descriptive statistics are reported as mean ± standard deviation (SD), median and interquartile range (IQR), or proportions with their 95 % CI.

The association between PELOD-2 score and death was assessed by comparing the PELOD-2 score between survivors and non-survivors with a Mann–Whitney test. We also tested this association, adjusting for the baseline risk (PELOD-2 score at day 1) and the daily change in PELOD-2 score using a logistic regression model [10].

*Discrimination* refers to the ability of the score to separate non-survivors from survivors across the whole group [19]. We calculated the area under the receiver operating characteristic curve (AUC) of the PELOD-2 scores with its 95 % CI, for each time point. It is usually considered that an AUC of 1, 0.90–0.99, 0.80–0.89, 0.70–0.79, 0.60–0.69, and <0.60 is considered to be perfect, excellent, very good, good, moderate, and poor, respectively [20].

The *calibration* was assessed by directly comparing the observed and customized predicted mortality across subcategories of risk. We employed the Hosmer–Lemeshow goodness-of-fit test, where a *p* value >0.05 indicates acceptable calibration [20].

All tests were two sided, with an alpha level of 0.05. All statistical analyses were performed with SPSS version 20 for Mac (SPSS, Chicago, IL, USA).

## Results

### Population

Over the 30 study days, 13,192 patients were admitted and hence eligible and 443 (3.4 %) critically ill children receiving at least one plasma transfusion were included. The center which included the largest number of patients contributed to 11.1 % (49/443) of the results. Two hundred and fifty-three patients were from Europe, 134 from North America, and 56 from other continents.

The median age and weight were 1 year (IQR 0.2–6.4) and 9.1 kg (IQR 4.0–21.0), respectively. Forty-three percent were males. The main reasons for admission to PICU were respiratory (32 %), cardiac surgery with bypass (30 %), elective surgery (24 %), septic shock (15 %), emergency surgery (13 %), cardiac non-surgical (11 %), renal failure (10 %), and hepatic failure (10 %). Forty-eight patients (11 %) were on extracorporeal life support, and 35 patients (8 %) were on continuous renal replacement therapy. The full demographic description is available elsewhere [11].

The primary indication for plasma transfusion was critical bleeding in 22 % of patients, minor bleeding in 21 %, planned surgery or procedure in 12 %, and high risk of postoperative bleeding in 11 %. No bleeding or planned procedures were reported in 34 % of patients.

Median length of mechanical ventilation was 5 days (IQR 1–16), and median PICU length of stay was 10 days (IQR 4–24). The median time to death was 6 days (IQR 1;17).

### PELOD-2 score

PELOD-2 score was collected in all patients on day 1 and in all surviving patients still in the PICU at posttransfusion days 2, 5, 8, and 12. There were no missing data.

**Fig. 1:**
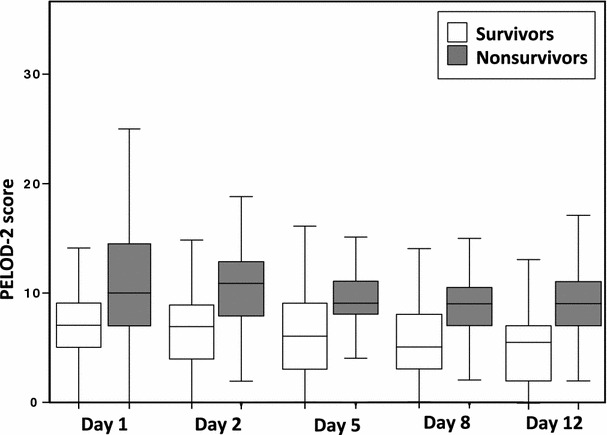
Boxplot of the PELOD-2 score on days 1, 2, 5, 8, and 12, among survivors (*white*) and non-survivors (*gray*). On each study day, PELOD-2 scores were significantly different between survivors and non-survivors

Median PELOD-2 scores were statistically different between survivors and non-survivors: 7 (5;9) versus 10 (7;15) on transfusion day 1 (*p* < 0.001), as well as on the other days (Table 1; Fig. 1).

**Table 1:** PELOD-2 score in survivors and non-survivors

Day	Survivors		Non-survivors		*p*
	Mean	Median (IQR)	Mean	Median (IQR)	
Day 1	6.7	7 (5;9)	11.2	10 (7;15)	<0.001
Day 2	6.5	7 (4;9)	11.0	11 (8;13)	<0.001
Day 5	6.1	6 (3;9)	9.2	9 (8;11)	<0.001
Day 8	5.6	5 (3;8)	8.8	9 (7;11)	<0.001
Day 12	5.1	5 (2;7)	8.5	9 (6;11)	<0.001

*PELOD-2 score* PEdiatric Logistic Organ Dysfunction-2 score, *IQR* interquartile range

The PELOD-2 score on day 1 was a significant prognostic factor: The odds ratio for death was 1.30 (95 % CI 1.22–1.39) for each PELOD-2 point. Similarly, the serial evaluation of the changes in the daily PELOD-2 scores from day 1, adjusted for baseline value (PELOD-2 at day 1), demonstrated a significant association with death, for each of the observation days (Table 2).

**Table 2:** Serial evaluation of the change in the daily PELOD-2 score from day 1, adjusted for baseline value (PELOD-2 score on day 1)

Variable	Odds ratio	95 % CI	*p* value
PELOD-2 score on day 1	1.30	1.22–1.39	<0.0001
Change in PELOD-2 score			
Day 1–day 2	1.33	1.21–1.47	<0.0001
Day 1–day 5	1.26	1.15–1.39	<0.0001
Day 1–day 8	1.28	1.15–1.43	<0.0001
Day 1–day 12	1.38	1.20–1.58	<0.0001

Odds ratio (OR) for death is provided with a 95 % CI. The cumulative OR of death can be calculated as follows: (OR of PELOD-2 score on day 1)^PELOD-2 score on day 1^ × (OR of change in score from day 1 to specific day)^PELOD-2 score on specific day – PELOD-2 score on day 1^. For example, for a child whose score is 10 on day 1 and 6 on day 12 (change in score −4), the OR for death would be 1.30^10^ × 1.38^−4^ = 3.80

*PELOD-2 score* PEdiatric Logistic Organ Dysfunction-2 score, *OR* odds ratio

### Discrimination

**Fig. 2:**
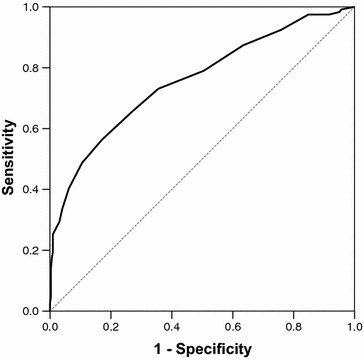
Receiver operating characteristic (ROC) curve of the PELOD-2 score on day 1. The area under the curve was 0.76 (95 % CI 0.71–0.81). The *dashed line* represents the reference line

The AUC was 0.76 (95 % CI 0.71–0.81, Fig. 2) for day 1 and 0.78, 0.74, 0.74, and 0.77, for days 2, 5, 8, and 12, respectively (Table 3). Adding the INR to the PELOD-2 score resulted in an AUC of 0.77 (95 % CI 0.71–0.83). The AUC on day 1 according to plasma transfusion indication and according to the reason for admission is presented in Tables 4 and 5, respectively.

**Table 3:** Performance of the PELOD-2 score according to study days

	Day 1	Day 2	Day 5	Day 8	Day 12
Number of patients	443	411	295	231	165
Mortality rate	119 (27 %)	98 (24 %)	67 (23 %)	51 (22 %)	41 (25 %)
PELOD-2 (median, IQR)	7 (5;10)	7 (5;10)	7 (4;9)	6 (3;9)	6 (3;9)
AUC (95 % CI)	0.76 (0.71–0.81)	0.78 (0.73–0.84)	0.74 (0.67–0.81)	0.74 (0.66–0.82)	0.77 (0.68–0.85)
Hosmer–Lemeshow test	*p* = 0.76	*p* = 0.63	*p* = 0.09	*p* = 0.30	*p* = 0.77

*PELOD-2 score* PEdiatric Logistic Organ Dysfunction-2 score, *IQR* interquartile range, *AUC* area under receiver the operating characteristic curve

**Table 4:** Performance of the PELOD-2 score on day 1, according to indications to plasma transfusion

	Critical bleeding	Minor Bleeding	Preparation^a^	Post-op risk of bleeding	No bleeding
Number of patients	99	94	52	47	151
Mortality rate	34 (35 %)	14 (16 %)	16 (32 %)	9 (19 %)	46 (31 %)
PELOD-2 (median, IQR)	8 (6–11)	7 (5–8)	7 (4–11)	7 (5–9)	7 (5–10)
AUC (95 % CI)	0.81 (0.72–0.91)	0.83 (0.70–0.96)	0.60 (0.44–0.76)	0.88 (0.74–1.00)	0.71 (0.62–0.80)
Hosmer–Lemeshow test	*p* = 0.42	*p* = 0.34	*p* = 0.26	*p* = 0.54	*p* = 0.58

*PELOD-2 score* PEdiatric Logistic Organ Dysfunction-2 score, *IQR* interquartile range, *AUC* area under receiver the operating characteristic curve

^a^Preparation refers to preparation for surgery or procedure

**Table 5:** Performance of the PELOD-2 score on day 1, according to patients’ reason for admission

	Respiratory	Cardiac surgery with bypass	Elective surgery	Septic shock	Renal failure
Number of patients^a^	143	133	107	65	45
Mortality rate	54 (38 %)	19 (14 %)	9 (8 %)	21 (32 %)	19 (42 %)
PELOD-2 (median, IQR)	8 (6–11)	7 (5–9)	6 (5–8)	8 (6–12)	8 (6–10)
AUC (95 % CI)	0.76 (0.68–0.85)	0.75 (0.62–0.88)	0.80 (0.62–0.97)	0.77 (0.66–0.89)	0.69 (0.53–0.85)
Hosmer–Lemeshow test	*p* = 0.99	*p* = 0.23	*p* = 0.17	*p* = 0.27	*p* = 0.39

*PELOD-2 score* PEdiatric Logistic Organ Dysfunction-2 score, *IQR* interquartile range, *AUC* area under receiver the operating characteristic curve

^a^Patients could be categorized into more than one reason of admission

### Calibration

The Hosmer–Lemeshow Chi-square value was 5.02 (*p* = 0.76) for PELOD-2 score on day 1. The results for the other time points, according to plasma transfusion indication, and according to the reason for admission are given in Tables 3, 4, and 5, respectively.

Adding the INR to the PELOD-2 score resulted in Hosmer–Lemeshow Chi-square value of 10.6 (*p* = 0.23).

## Discussion

Our results indicate that in a subpopulation of critically ill children requiring plasma transfusions, the PELOD-2 score has an acceptable performance. These results of performance of the PELOD-2 scores are observed with the performance both according to the indications to plasma transfusion (Table 4) and according to the 5 days of the PELOD-2 scores (Tables 2, 3).

The PELOD-2 score seems to have a lower, although acceptable discrimination power compared to a general PICU population, where it had an excellent discrimination power, based on an AUC of 0.93. As the PELOD-2 score has been advocated to be “used as a surrogate outcome measure in randomized clinical trials” [7, 8], many recent trials have used these scores to assess patients [13–18]. As these studies enrolled only specific PICU subpopulations, their results should be interpreted cautiously, in light of our findings. Similar caveats have been made for adult organ dysfunction scores regarding their use in specific subpopulations [9].

The lower discrimination of the PELOD-2 score in a subset of critically ill children transfused with plasma might be explained by different observations. First, our population was sicker than the initial PELOD-2 study, with a mortality rate of 26.9 versus 6.0 %. The PICU length of stay was also longer (10 vs. 2 days), as the case-mix was different: In our population compared to the PELOD-2 population, the reasons for admission were less frequently respiratory (32 vs. 47 %, *p* < 0.001) and more frequently cardiovascular (64 vs. 19 %, *p* < 0.001) or hepatic (10 vs. 1 %, *p* < 0.001) [8]. Moreover, in the study of Pollack et al. (10,078 patients from U.S. PICUs), the mortality rate was 2.7 %, with a median age of 3.7 years and a median hospital length of stay of 4.9 days (PICU length of stay was not provided) [21]. The fact that all patients received plasma transfusions might also explain this lower discrimination, as observational studies have suggested that plasma transfusions were independently associated with increased risk of morbidity and mortality [9, 22–24], but neither coagulopathy nor plasma transfusions are items of the PELOD-2 score. Third, except for mechanical ventilation, PELOD-2 score does not take into account the support that can be offered for each organ, such as vasoactive drugs, continuous renal replacement therapies, or extracorporeal life support (ECLS). Given that the mortality rate associated with ECLS is close to 50 % [25, 26], one could hypothesize that a lower discrimination may be due at least partly to the fact that PELOD-2 score does not take this variable into account. Similarly, the PELOD-2 score does not incorporate coagulopathy, which might be associated with increased risk of bleeding or disseminated intravascular coagulopathy, which are also known to be associated with increased mortality [27].

As observed in the validation study, severity of illness is a major component to evaluate the performance of the daily PELOD-2 scores [10]. Therefore, in this study, the serial evaluation of the change in the daily PELOD-2 score has to be adjusted to the baseline value (PELOD-2 score on day 1). Our results indicate that changes in the PELOD-2 score over time are also associated with changes in the probability of death. These are important findings, as they validate the use of sequential measuring of the PELOD-2 score to assess clinical outcome. These results also highlight the relationship between organ failure and death.

Some limitations must be recognized. First, our results are only applicable to critically ill children requiring plasma transfusions and not to other PICU subpopulations. Second, our study days were not days after admission but days after plasma transfusion. It is of note, however, that the median length of PICU stay before the first plasma transfusion was 1 day. Third, although scores have classically been evaluated by their association with mortality, they might still adequately describe the clinical situation related to organ failure, as it has solid physiological basis and is clinically meaningful. Therefore, the ability of the PELOD-2 score to describe organ failure and its change over time might be more important that its ability to predict death. Fourth, additional variables to the PELOD-2 score could have been evaluated. Unfortunately, some interesting variables, such as platelet count, were not available in our database. However, adding the INR to the PELOD-2 score did not improve its performance.

## Conclusions

In a subpopulation of critically ill children requiring plasma transfusion, the PELOD-2 score has a lower but acceptable discrimination than in an entire population. Although using this score as an outcome in a RCT seems reasonable, it should be interpreted cautiously.

## Abbreviations

AUC: area under the receiver operating characteristic curve
CI: confidence interval
ECLS: extracorporeal life support
FiO_2_: inspired fraction of oxygen
IQR: interquartile range
MODS: multiple organ dysfunction syndrome
OR: odds ratio
SD: standard deviation
PaO_2_: partial pressure of arterial oxygen
PaCO_2_: partial pressure of arterial carbon dioxide
PELOD-2: PEdiatric Logistic Organ Dysfunction-2 score
PICU: pediatric intensive care unit
